# Influence of High Shear Dispersion on the Production of Cellulose Nanofibers by Ultrasound-Assisted TEMPO-Oxidation of Kraft Pulp

**DOI:** 10.3390/nano2030286

**Published:** 2012-09-10

**Authors:** Eric Loranger, André-Olivier Piché, Claude Daneault

**Affiliations:** 1 Lignocellulosic Material Research Centre, Université du Québec à Trois-Rivières, 3351 Des Forges, Trois-Rivières, Québec G9A 5H7, Canada; Email: andre-olivier.piche@uqtr.ca (A.-O.P.); claude.daneault@uqtr.ca (C.D.); 2 Canada Research Chair in Value-added Paper, 3351 Des Forges, Trois-Rivières, Québec G9A 5H7, Canada

**Keywords:** Kraft pulp, ultrasound, TEMPO-oxidation, nanocellulose, consistency, high-shear dispersion, stator-rotor gap, recirculation rate, pH, rheological behavior

## Abstract

Cellulose nanofibers can be produced using a combination of TEMPO, sodium bromide (NaBr) and sodium hypochlorite, and mechanical dispersion. Recently, this process has been the subject of intensive investigation. However, studies on the aspects of mechanical treatment of this process remain marginal. The main objective of this study is to evaluate the high shear dispersion parameters (e.g., consistency, stator-rotor gap, recirculation rate and pH) and determine their influences on nanocellulose production using ultrasound-assisted TEMPO-oxidation of Kraft pulp. All nanofiber gels produced in this study exhibited rheological behaviors known as shear thinning. From all the dispersion parameters, the following conditions were identified as optimal: 0.042 mm stator-rotor gap, 200 mL/min recycle rate, dispersion pH of 7 and a feed consistency of 2%. High quality cellulose gel could be produced under these conditions. This finding is surely of great interest for the pulp and paper industry.

## 1. Introduction

To counterbalance the shrinking market of consumer paper products, the pulp and paper industries are intensifying their research efforts to diversify the use of wood raw material. Strategies to produce value-added products from lignocellulosic biomass have emerged [1], particularly in the field of development, production and application of nanocellulose [2,3]. Literature has shown that cellulose nanofibers can be produced by an oxidation system involving the use of a catalyst of TEMPO, sodium bromide (NaBr) and sodium hypochlorite (NaOCl), followed by mechanical dispersion [4,5,6,7,8]. Our recent studies indicate that the oxidation efficiency can be significantly improved with 15% to 30% increase in carboxylate content [9,10,11,12] when the oxidation was conducted in the presence of ultrasonic cavitation.

Despite numerous studies [4,5,6,7,8,9,10,11,12] on the preparation of cellulose nanofibers, the mechanism of mechanical dispersion has rarely been investigated. The use of a blender does generate great shear rate, but higher shear rate mixers might be more efficient in nanofiber dispersion and are available on the market [13]. High shear rate equipment such as those provided by IKA Works Inc. (USA) are used in the food [14] and pharmaceutical fields [15]. Here are a few examples of applications: homogenization, dispersion, emulsification, grinding, dissolving, chemical reaction and cell disruption [13].

Some researchers [16,17] examined the correlation between the quality of nanocellulose dispersion and rheological measurements. The following exponential relationship between the shear rate (*γ*) and shear stress (*σ*) has been proposed [16] and expressed in Equation (1): where *K* is the viscosity coefficient and *n*, the flow behavior index. By definition, shear thinning is when the viscosity of a fluid decreases with increasing shear rate while shear thickening is the opposite. When using Equation (1), if *n* is equal to 1, then the fluid is Newtonian. When it is less than 1, it indicates a shear thinning fluid. In contrast, when *n* is greater than 1, it indicates a shear thickening fluid. The reader should note that Equation (1) is not universal but can be successfully applied to a wide range of fluids, which is the case in our study. Rheological measurements can be associated to particle morphology and aggregation potential.


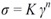
(1)

The main objective of this study is to examine the influence of high shear dispersion parameters on the characteristics of nanocellulose produced by TEMPO-mediated oxidation of Kraft pulp in the presence of ultrasound waves.

## 2. Results and Discussion

Previously, we have shown that it is possible to scale up the production of oxidized cellulose fibers by performing the TEMPO-mediated oxidation in a flow-through sonoreactor system [18]. The optimal ultrasonic conditions determined in our previous research [18] on production of oxidized cellulose with carboxylate content of 935 mmol/kg, were applied. The carboxylate content of cellulose produced in our experiments, which is superior to that indicated in the literature, indicates the efficiency of ultrasound conditions of our experiments. As a reference, the carboxyl density of unoxidized bleached Kraft pulps is known to be less than 100 mmol/kg [19]. According to Saito *et al.* [5], the oxidized fibers with carboxylate content less than 600 mmol/kg were mostly unfibrillated by the mechanical treatment and maintained their original fibrous morphologies. Since our oxidized pulp carried a carboxylate content which is much higher than that reported by Saito *et al.* [5], these oxidized fibers should be readily dispersed.

### 2.1. Experimental Error

**Figure 1:**
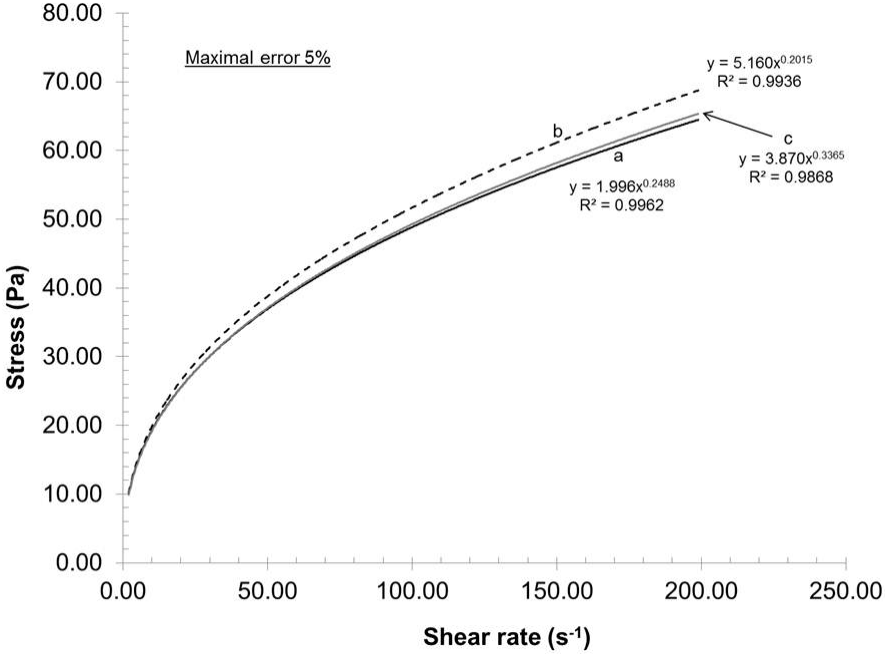
Experimental error assessment.

To evaluate the experimental error of the mechanical dispersion and rheological measurements, three trials (a, b, c) were executed using pulps having the same carboxylate content and under identical dispersion conditions (recycle rate, gap, pH and consistency). The experimental error of each trial was then evaluated using a rheometer (ATS RheoSystems) and the curve fitted results are illustrated in Figure 1. As the correlation coefficient is close to 1, the fitted data trends are identical to experimental data trend; all experimental data points are omitted for the purpose of clarity.

Figure 1 shows that curves a and c were practically identical, whereas the stress of curve b was in general larger than those of curves a and c. As the gap between the curves increased with increasing shear rate, the maximal experimental error will be reported in this study. From these three experiments, the maximal error achieved was 5% in stress. As a result, based on this value, the entire process can be considered as highly reproducible and the curves reported in this study as representative.

### 2.2. Optimization of Mechanical Dispersion of Oxidized Fibers Using Rheological Curves

#### 2.2.1. Influence of Consistency

**Figure 2:**
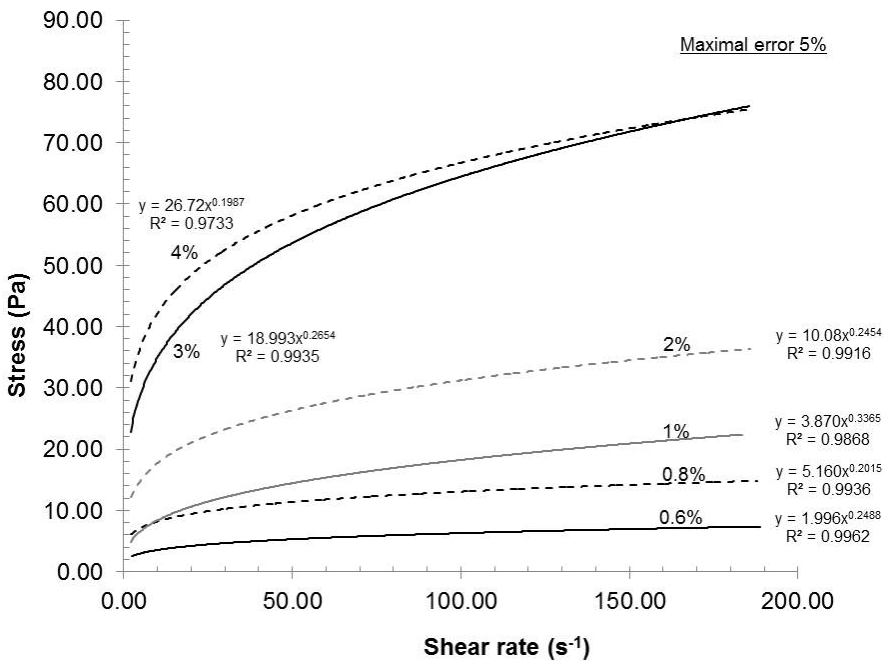
Effect of consistency on the rheological evolution of the gels.

**Figure 3:**
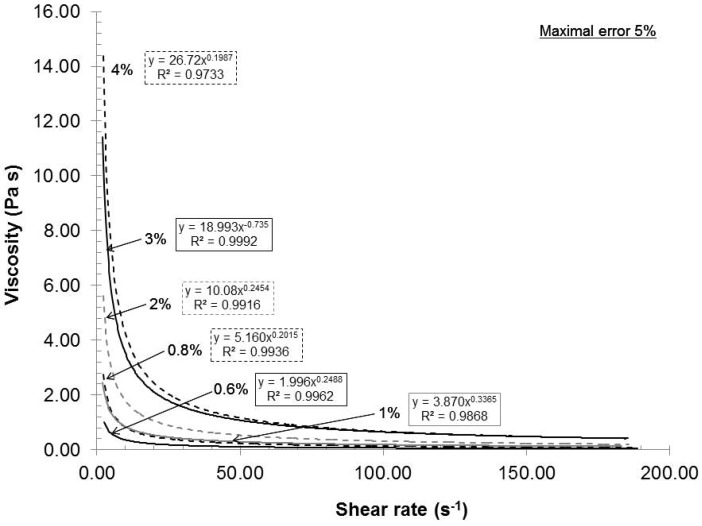
Effect of consistency on the viscosity of the gels.

The feed consistency was varied to access its influence on mechanical dispersion of the oxidized pulp, which has a carboxylate content of 935 mmol/kg. Figure 2 presents the relationship between the consistency and the rheological behavior of the gels under these conditions: Rotor-stator gap 0.042 mm, recycle rate 200 mL/min and pH 7. As seen in Figure 2, the gels exhibited shear thinning rheological behaviors at all consistencies. Such a result is not uncommon, because the gels are composed of nanofibers and fibers (microfibers) of cylindrical shape with network capabilities (hydrogen bond). When the shear rate increased, the fiber and nanofiber network slowly broke up into individual entities and they oriented themselves in a parallel formation, thus reducing the final viscosity of the gels (Figure 3). Such behavior is frequently observed in the dispersion of polymer. The polymer elements are similar to the oxidized fibers in shape [20]. These results indicated that when the shear rate increased, the resulting viscosity decreased while the applied stress increased.

**Figure 4:**
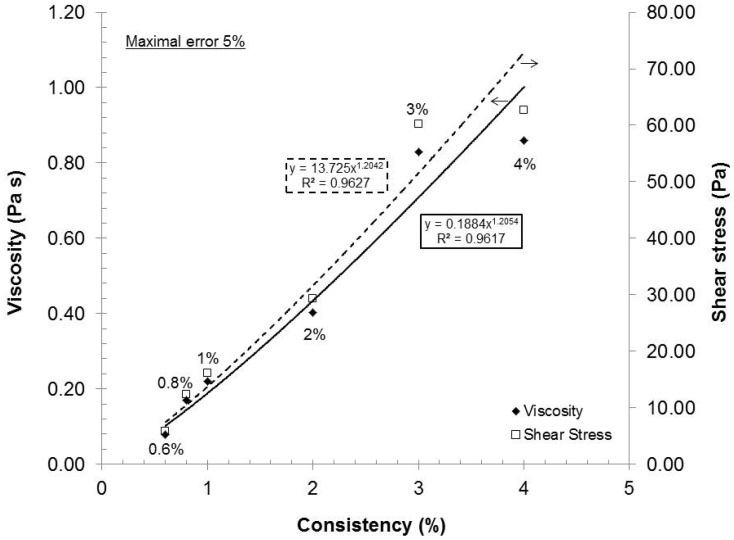
Gel viscosity evolution trend for the various consistencies at shear rate of 73.42 s^−1^.

As the consistency increased (increased fiber concentration), the statistical probability of contact, initial viscosity or stress also augmented. The final stress can then be correlated to the network and gel strength or dispersion quality. If we chose an arbitrary shear stress (e.g., 73.42 s^−1^) in Figure 2 and Figure 3, and compare the resulting viscosities in Figure 4, we can conclude that the rheological response of stress or viscosity to the shear rate does not follow a true linear trend but rather a subtle power function. Such behavior is expected as the statistical probability of collision between fibers increases exponentially to their number. This characteristic has been observed for TEMPO-oxidized fibers [16]. From a gel quality standpoint, 4% consistency was the best option. But, at this consistency, the pumping needs a lot of power and, therefore, great energy loss is transferred as heat in the gel. The resulting heating then requires more cooling to maintain the experimental temperature. In this aspect, further laboratory experiments were made at 2% consistency.

#### 2.2.2. Influence of Stator-Rotor Gap

**Figure 5:**
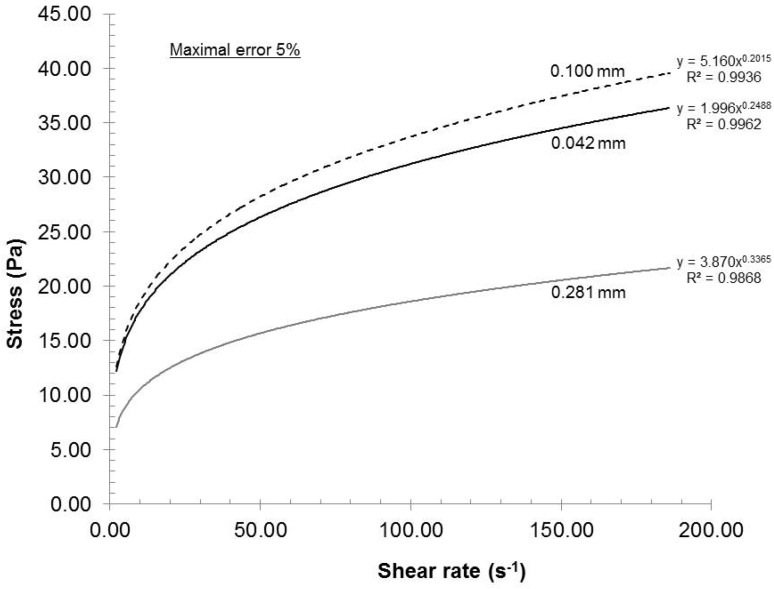
Effect of stator-rotor gap on the rheological evolution of the gels.

In a rotor and stator configuration, a reduced gap will develop higher shear force that should result in an increase in stress on individual fibers. Figure 5 presents the rheological curves as a function of gap at 2% consistency, 200 mL/min recycle rate and pH 7. In this figure, we can clearly identify two groups. The biggest gap had resulted in a network with lower stress capacity as compared to the other two gap settings. However, within the maximal error of 5%, the curves for gaps of 0.042 and 0.1 mm were similar. At maximal motor speed, the maximal admissible gap, the gap before losing dispersion similarity to 0.042 mm, will be found between 0.1 and 0.281 mm. Additional reduction in gap will not increase the dispersion quality and, in fact, would increase the heat generated by friction, causing useless energy loss. From a laboratory stand point, the 0.042 mm gap is identified as optimal but further investigation will be required if thermal energy is to be taken into account.

#### 2.2.3. Influence of Recirculation Rate

**Figure 6:**
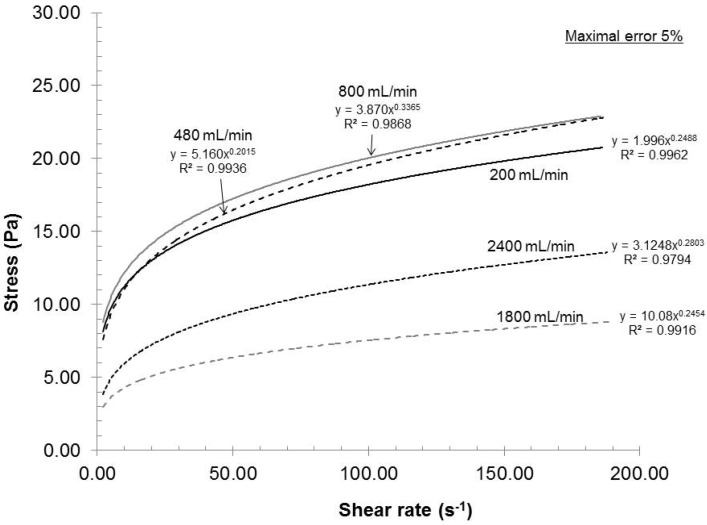
Effect of recirculation rate on the rheological evolution of the gels.

In the dispersion unit, a volume of 2 L of oxidized pulp suspension circulated in a closed loop for a total time of 1 h. The recirculation rate is, therefore, directly related to the amount of passes or cycles that the pulp will undergo. A low recycle rate will increase the retention time in the dispersion unit, resulting in a lower number of passes. In contrast, a high recycle rate will reduce the retention time and increase the number of passes. Figure 6 presents the effect of recycle rate on the dispersion quality at 2% consistency, a rotor-stator gap of 0.042 mm and pH 7.

From the results shown in Figure 6 and considering the statistical error, the effect of the retention time was not evident for flow rates between 200 and 800 mL/min. However, as the flow rate increased, the dispersion quality was drastically reduced. Consequently, the condition of fewer passes at high retention time gave better gel quality than those with a greater number of passes at lower retention time. This effect is similar to that of a thermomechanical pulp refiner where the fiber quality is greatly influenced by the feed rate [21]. A recycle rate of 200 mL/min is then preferable for laboratory experiment as well as for the eventual industrial production because the dispersion stage would require smaller equipment, thus smaller investment.

#### 2.2.4. Influence of Dispersion pH

**Figure 7:**
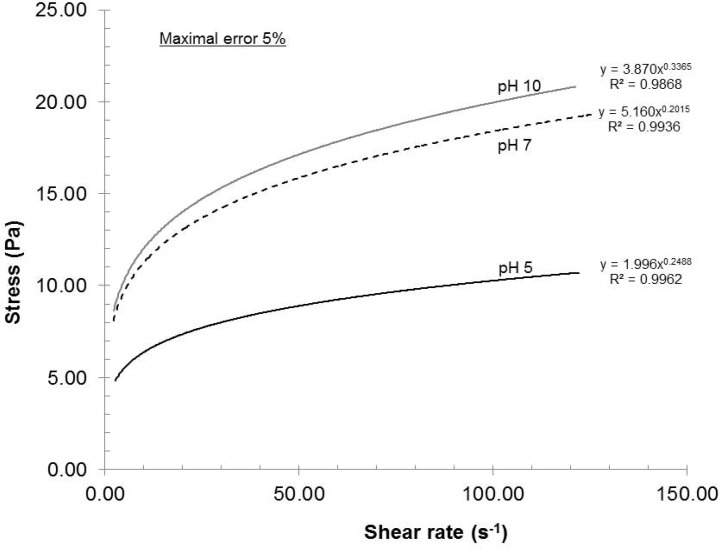
Effect of dispersion pH on the rheological evolution of the gels.

As reported in References [4] and [16], the dispersion pH greatly influences the surface charge of the nanofibers. With low pH, the carboxyl groups become protonated and lose their charge. Consequently, the beneficial electrostatic repulsion is greatly diminished and will show up in the rheological profile (Figure 7).

The rheological profiles shown in Figure 7 are clear examples of this situation. The dispersion quality was the lowest at pH 5 and the highest at pH 10, meaning that a minimum surface charge was observed at pH 5 and a maximum value was noted at pH 10. However, the difference in surface charge between pH 7 and pH 10 was almost within the experimental error. The pH 5 clearly gave the worst quality measured. Although pH 10 yielded the best results, a dispersion pH of 7 should be chosen in order to prevent hydrolysis of pulp when the sample is stored at pH 10.

## 3. Experimental Section

### 3.1. Materials

A commercial never-dried bleached Kraft wood pulp (carboxyl content: 53 mmol/kg) was provided by Fraser Paper (Thurso, Canada) and used in this study as native cellulose fibers. Free radical 4-acetamido-TEMPO (2,2,6,6-tetramethylpiperidin-1-oxyl) was purchased from Sigma-Aldrich (Canada) and sodium bromide from Fisher Scientifics (Canada). All chemicals were ACS reagent grade. Sodium hypochlorite at 6.0% was purchased at the supermarket.

### 3.2. TEMPO-Mediated Oxidation under Ultrasonic Cavitation

**Figure 8:**
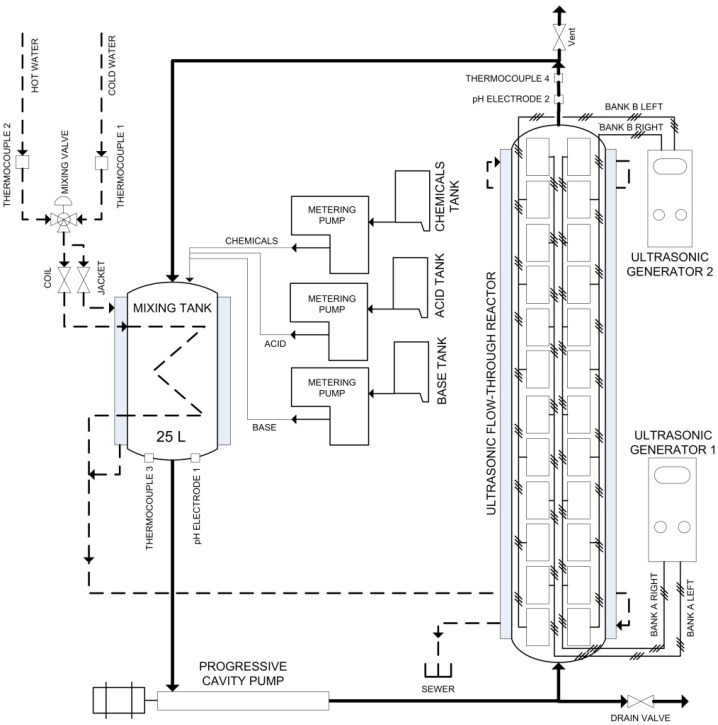
Schematic diagram of the large-scale flow-through sonoreactor.

The oxidation process was conducted in a 45 L flow-through sonoreactor (semi-continuous mode) with a nominal input power capacity of 2000 W (1.23 W/cm^2^ or 262 W/L at maximum applied intensity, Figure 8).

The concept of sonoreactor has been previously discussed in detail [18]. Before oxidation, 400 g of dried bleached pulp were pre-soaked for two days in 40 L of distilled water at room temperature. The soaked pulp was disintegrated for 10 min in a laboratory disintegrator to obtain a uniform fiber suspension of about 1% consistency. To the cellulose suspension we added 0.1 mmol/g of dry cellulose of 4-acetamino-TEMPO, 3.1 mmol/g of dry cellulose of NaOCl and 0.61 mmol/g of dry cellulose of NaBr. The NaOCl reagent was introduced using a peristaltic pump during the first 30 min. The NaBr, a co-catalyst, also increased the reaction velocity. However, a high amount of NaBr can decrease the ability to detect the effect of ultrasound because of its doping effect on the reaction system [11]. The other reaction conditions were pH 10.5, 25 °C and 90 min. The acoustic frequency used was 170 kHz (125 W) which was found earlier to be optimum [11,18]. The reaction was stopped after 90 min by adding 1 L of H_2_O_2_ (1%), and the final pH of the suspension was adjusted to 7 by adding 0.5 M NaOH or HCl as required. Finally, the oxidized fibers suspension obtained was filtered and thoroughly washed with distilled water on a filter paper, and stored at 4 °C to be used for further analysis.

### 3.3. Measurement of Carboxylate Group Content

Carboxylic acid groups generated during oxidation were determined with a Dosimat 765 titrator from Metrohm (USA) and a conductivity meter (Orion 3Star) from Thermo (USA). The carboxylate content of oxidized pulp was measured using conductimetric titration [22]. Briefly, a sample (2 g, o.d. basis) of oxidized fibers was first washed twice—using 250 mL 0.1 N HCl—to convert the carboxylate groups into free carboxyl. The treated pulp was thoroughly washed twice with distilled water (2 × 500 mL) to remove any excess acid. The washed sample (acidified pulp) was dispersed in 450 mL of 0.001 N NaCl with an addition of 5.0 mL of 0.1 N HCl. The suspension was then titrated with 0.1 N NaOH. After the titration, the sample was thickened by filtration and dried at 105 °C to obtain its o.d. weight for the calculation of carboxylate content.

### 3.4. High Shear Dispersion

**Figure 9:**
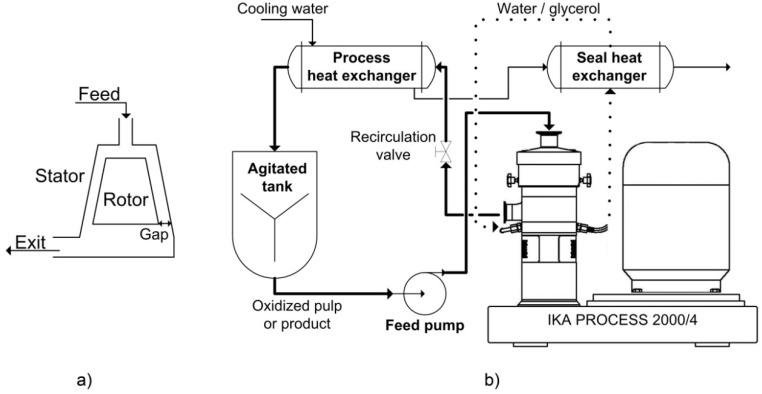
(**a**) Schematic representation of the operation principle; (**b**) Schematic diagram of the colloid milling apparatus (MK 2000/4) from Process IKA Works, Inc.

High shear dispersion was performed in a wet colloid milling apparatus (MK 2000/4) from IKA Works, Inc. (USA). The oxidized pulp suspension (2 L) was pumped from an agitated tank between a conical rotor and a stator in the MK mill as shown in Figure 9a. The gap between the rotor and the stator is adjustable to achieve various shear rates. Figure 9b shows a schematic of the entire process. The residence time in the MK mill is controlled manually by the recirculation valve and the feed pump speed. The pulp or product then passes through a heat exchanger, cooled with tap water to maintain the temperature at 25 °C. The same cooling water was used to remove heat from the bearing seal loop to ensure proper operation of the mill. In our work, the mill was operated in closed loop for a given amount of time (1 h); in a typical industrial application, the process would be continuous. The experimental conditions explored were: 0.6% to 4% consistency of the feed pulp, 0.042 to 0.281 mm for the stator-rotor gap, 200 to 2400 mL/min for the recirculation rate and at pH 5, 7 and 10. The normal operating conditions of this laboratory setup were: 2% consistency, 0.073 mm gap, 200 mL/min recirculation rate, 25 °C, pH 7 and 1 h.

### 3.5. Rheological Measurements

The quality of the dispersion was measured by rheological measurement by means of a STRESSTECH rheometer (ATS RheoSystems), equipped with a cone-plate measurement head (40 mm diameter, 4° angle). With this measurement head, shear rate from 2 to 200 s^−1^ were explored upstream and downstream. Sample stress and viscosity were then related to the shear rate to study the rheological behavior such as shear thinning, shear thickening or even Newtonian fluid. The instrument used an automatic real time inertia compensation to make high precision measurements and it was also equipped with a patented quantitative normal force capability. All experiments were carried out at room temperature (23 °C).

## 4. Conclusions

Since we have determined that the exponent *n* of the exponential relation (1) is always below 1, all nanofibers gels produced in this study exhibit rheological behavior known as shear thinning. The final stress can then be correlated to the network and gel strength or dispersion quality.

Consistency optimization reveals that the rheological response of stress or viscosity to the shear rate does not follow a linear trend but rather a power function. From a gel quality standpoint, 4% consistency is the best condition; but 2% was chosen for laboratory considerations. The maximal admissible gap is between 0.1 and 0.281 mm; additional reduction does not increase the dispersion quality. From a laboratory standpoint, the 0.042 mm gap is considered optimal; further investigation will be required if energy is to be taken into consideration. A low recycle flow rate (fewer passes at high retention time) has resulted in higher gel quality as compared to a high recycle flow rate (more passes at lower retention time). A recycle rate of 200 mL/min is preferable for laboratory experiments and for eventual industrial production as the dispersion step would require smaller investment. Since the variation of dispersion pH between pH 7 and 10 was determined to have no influence on the dispersion quality and lower pH of 5 is detrimental to the dispersion quality, the dispersion pH of 7 was chosen as optimal.

Hence, based on the rheological measurements and dispersion conditions, the followings operating conditions were identified as optimal in our laboratory apparatus: 0.042 mm gap, 200 mL/min recycle rate, dispersion pH of 7 and a feed consistency of 2%. These experimental values can serve as the basis for scaling up the production of nanocellulose fibers at an industrial level.
